# CD56^+^CD57^+^ infiltrates as the most predominant subset of intragraft natural killer cells in renal transplant biopsies with antibody-mediated rejection

**DOI:** 10.1038/s41598-019-52864-5

**Published:** 2019-11-12

**Authors:** Hey Rim Jung, Mi Joung Kim, Yu-Mee Wee, Jee Yeon Kim, Monica Young Choi, Ji Yoon Choi, Hyunwook Kwon, Joo Hee Jung, Yong Mee Cho, Heounjeong Go, Sang-Yeob Kim, Yeon-Mi Ryu, Yun Jae Kim, Young Hoon Kim, Duck Jong Han, Sung Shin

**Affiliations:** 1 Division of Kidney and Pancreas Transplantation, Department of Surgery, Asan Medical Center, University of Ulsan College of Medicine, Seoul, Korea; 2 Department of Pathology, Asan Medical Center, University of Ulsan College of Medicine, Seoul, Korea; 3 Department of Medical Science, Asan Medical Institute of Convergence Science and Technology, Asan Medical Center, University of Ulsan College of Medicine, Seoul, Korea; 4 Department of Convergence Medicine, Asan Medical Center, University of Ulsan College of Medicine, Seoul, Korea; 5 Asan Institute for life sciences, Asan Medical Center, Seoul, Korea

**Keywords:** Allotransplantation, Translational research

## Abstract

Little is known about the characteristics and clinical implications of specific subsets of intragraft natural killer (NK) cells in kidney transplant recipients. We analyzed 39 for-cause renal transplant biopsies performed at our center from May 2015 to July 2017. According to histopathologic reports, 8 patients (20.5%) had no rejection (NR), 11 (28.2%) had T cell-mediated rejections (TCMR) only, and 20 (51.3%) had antibody-mediated rejection (ABMR). NK cells were defined as CD3^–^CD56^+^ lymphocytes that are positive for CD57, CD49b, NKG2A, or KIR. The density of NK cells was significantly higher in the ABMR group (2.57 ± 2.58/mm^2^) than in the NR (0.12 ± 0.22/mm^2^) or the TCMR (0.25 ± 0.34/mm^2^) group (*P* = 0.002). Notably, CD56^+^CD57^+^ infiltrates (2.16 ± 1.89) were the most frequently observed compared with CD56^+^CD49b^+^ (0.05 ± 0.13), CD56^+^NKG2A^+^ (0.21 ± 0.69), and CD56^+^KIR^+^ (0.15 ± 0.42) cells in the ABMR group (*P* < 0.001). Death-censored graft failure was significantly higher in patients with NK cell infiltration than those without (Log-rank test, *P* = 0.025). In conclusion, CD56^+^CD57^+^ infiltrates are a major subset of NK cells in kidney transplant recipients with ABMR and NK cell infiltration is significantly associated with graft failure post-transplant.

## Introduction

Antibody-mediated rejection (ABMR) is the leading cause of late allograft failure after solid organ transplantation and contributes to two-thirds of kidney transplant failure^1,2^. Accordingly, recent reports showed that donor-specific antibodies (DSAs) are an important prognostic factor for kidney allograft survival^3,4^. Although kidney allograft survival has significantly improved with the introduction of potent immunosuppression regimens, current immunosuppressants mainly target T cells to prevent T cell-mediated rejection (TCMR). Due to the important role of DSAs in ABMR, several therapies have been developed to deplete B cells and DSAs and to inactivate plasma cells; however, most of these therapies are limited in preventing or treating ABMR^5–7^. Therefore, novel strategies that prevent and treat ABMR need to be developed in order to further improve clinical outcomes after kidney transplantation.

NK cells are paradoxically involved in both graft acceptance and dysfunction through their influence on related immune pathways^8–10^. Recent reports utilized microarray transcriptomic data to show that NK cells play a central role in the pathophysiology of ABMR and graft failure after kidney transplantation^11–13^. Nevertheless, the exact mechanism of NK cells and NK activation markers for ABMR are yet to be determined. While first evaluated as a NK cell marker, CD57 has been almost considered as a marker of replicative senescence on T cell^14^. It has been, however, reported that acquisition of CD57 reflects toward a higher cytotoxic capacity, greater responsiveness to signaling and decreased responsiveness to cytokines rather than being a marker of anergy or immunosenescence^15,16^. It was also demonstrated that human cytomegalovirus drives expansion of NKG2C^ +^ NK cells and that these cells preferentially acquire CD57^17,18^. On the other hand, CD94/NKG2A heterodimer and the killer immunoglobulin-like receptor (KIR) family inactivate NK cells interacting with HLA-E and distinct sets of classical HLA class I molecules^19–21^. So far, it has been unrevealed which subsets of NK cells are involved in ABMR after kidney transplantation. In this study, we characterized the specific subsets of intragraft NK cells in human kidney transplant biopsies from patients with ABMR by using multiplex immunohistochemistry and investigated clinical implications of each NK cell subset with respect to graft survival.

## Materials and Methods

### Patients

We analyzed the for-cause kidney allograft biopsies that were performed at Asan Medical Center from May 2015 to December 2016. All consenting kidney transplant recipients who had a for-cause biopsy for clinical indication (proteinuria or deterioration in function) in five years after kidney transplantation were enrolled. Pediatric renal transplant or multiple solid organ transplant recipients were excluded in this study. Written informed consent was achieved from all recipients. The study was approved by the institutional review board of Asan Medical Center (Approval Number: 2015-0758). Written informed consents from kidney donors were waived because it was not available to get an informed consent at the time of a for-cause biopsy. The same institutional review board that approved this study also approved the lack of informed consent of kidney donors.

Baseline characteristics were recorded and analyzed after receiving approval from the institutional review board. No organs/tissues were procured from prisoners. All the living donor nephrectomy was performed at Asan Medical Center while kidneys from deceased donors were procured at several centers in South Korea; one at Asan Medical Center, three at Ajou University Hospital, one at Ewha Womans University Mokdong Hospital, two at Seoul St. Mary’s Hospital, one at Samsung Medical Center, two at Seoul National University Hospital, and two at Severance Hospital. Every deceased donor was strictly managed by a government organization (KONOS, Korean Network for Organ Sharing) without any organ trade or illegal distribution.

All experiments were performed in accordance with relevant guidelines and regulations.

### Immunosuppression

As an induction regimen, basiliximab and rabbit anti-thymocyte globulin (Thymoglobulin®, Genzyme, Cambridge, MA, USA) were administered in thirty-six and three patients, respectively. The maintenance immunosuppressants consisted of a calcineurin inhibitor (tacrolimus or cyclosporine), mycophenolic acid, and prednisolone.

### Histopathology

Every biopsy had at least one artery and seven glomeruli, which was appropriate for the diagnosis and classification of rejection. Each biopsy was stained with periodic acid-Schiff (PAS), hematoxylin and eosin (H&E), Masson trichrome, and Jones-methenamine silver for interpretation. C4d immunohistochemistry (1:100, rabbit polyclonal; Cell Marque, Rocklin, CA, USA) was performed on paraffin-embedded, formalin-fixed specimen using the Ventana Medical Systems, Tucson, AZ, USA) according to the manufacturer’s protocol^22^. Every allograft biopsy specimen was evaluated for histologic characteristics according to the Banff 2015 criteria^23^ by two renal pathologists (YM Cho and H Go), and biopsies that lacked histological disease features were diagnosed as normal. C4d staining ≥10% was interpreted as positive. Interstitial fibrosis and tubular atrophy (IFTA) was defined as ci score >0. Inflammation in IFTA areas (i-IFTA) was scored according to the Banff 2017 criteria^24^ by the two pathologists (YM Cho and H Go).

### Opal multiplex immunohistochemistry

Fully-automated seven-color immunohistochemistry method of Opal multiplex staining was carried out with Opal™ multiplex kit (PerkinElmer^®^) by using Leica Bond Rx™ Automated Stainer (Leica Biosystems) at the Optical Imaging Core Laboratory in Asan Institute for Life Sciences in Asan Medical Center. Formalin-fixed paraffin-embedded (FFPE) tissue blocks from selected H&E biopsy slides were obtained. The sample slides were baked at 60 °C for four hours in a dry oven and then transferred to the Leica Bond Rx™ Automated Stainer. First, the slides were rinsed with 100% xylene for ten minutes, which was repeated three times to deparaffinize; sections were then rehydrated in a series of diluted alcohols to distilled water. Antigen retrieval was performed in citrate buffer (pH 6.0) using microwave treatment (MWT). Subsequent Opal staining of each antigen (CD3, CD56, CD57, CD49b, NKG2A, KIR) was carried out by washing and blocking with 3% H_2_O_2_ blocking solution, followed by antibody diluent. The first primary antibody was incubated for one hour at optimized concentrations–CD3 (Dako, rabbit polyclonal, 1:100), CD56 (Leica Biosystem, mouse monoclonal, clone CD564, 1:100), CD57 (Thermofisher, mouse monoclonal, clone NK1, 1:100), CD49b (GeneTex, mouse monoclonal, clone 16B4, 1:50), NKG2A (Abcam, rabbit polyclonal, 1:100), KIR (LSBio, rabbit polyclonal, 1:200), followed by Opal HRP polymer and one of the Opal fluorophores (CD3: Opal 540, CD56: Opal 570, CD57: Opal 650, CD49b: Opal 690, NKG2A: Opal 620, KIR: Opal 520) to visualize antigens. Afterward, antibody stripping was performed by placing the slides in citrate buffer (pH 6.0) and heating them using MWT; as such, individual antibody complexes were stripped after each round of antigen detection. These steps were repeated from blocking step to antibody stripping step until all antigens were detected by using a different Opal fluorophore for each target. After the final stripping step, nuclei were stained with DAPI included in the Opal™ multiplex kit (PerkinElmer^®^), and cover-slipped by using HIGHDEF® IHC fluoromount (ADI-950-260-0025, Enzo, USA)^25,26^.

### Image acquisition and quantitative image analysis

Multiplex-stained FFPE tissue slides were scanned, and the multispectral images were captured for all target markers through Vectra® Polaris Automated Quantitative Pathology Imaging System (PerkinElmer^®^). Using the Region of Interest (ROI) Tool in the Phenochart software, ROIs were drawn in each scanned tissue slide as many as possible to cover most of the whole tissue; as a result, each tissue had different numbers of ROI due to differences in sample size. To establish a multispectral library containing the emitting spectral peaks of all fluorophores, the spectral information was reliably unmixed and quantitated for correct examples of each fluorophore emission spectra^25^. Accordingly, the individually stained sections (CD3: Opal 540, CD56: Opal 570, CD57: Opal 650, CD49b: Opal 690, NKG2A: Opal 620, KIR: Opal 520) were used to confirm the library for multispectral analysis. After visualizing the images, we used an analysis algorithm in InForm 2.4 software to create spectral composite images and analyzed the exported data and images with Spotfire software (TIBCO Software Inc.). The nucleus of each cell was identified by detecting DAPI. Based on DAPI visualization, all cases that were CD3^−^CD56^+^ and positive for at least one of the other surface markers (CD57, CD49b, NKG2A, and KIR) were considered as infiltrated NK cells. CD3^+^CD56^−^ infiltrates were identified as T cell whereas CD3^+^CD56^+^ as NKT cells. The infiltrated NK cells were counted by using the analysis software, and their density on each slide was calculated by dividing their absolute (total) number by the total area of ROI. If infiltrates were positive for more than three out of four NK markers (CD57, CD49b, NKG2A and KIR), they were counted repeatedly and divided by the number of positive markers to belong to each subpopulation of intragraft NK cells. The total area of ROI also was calculated by multiplying the total number of ROI in each slide by 0.69 mm^2^ (one ROI: 1 field = 0.69 mm^2^, 1 field = 2,628,288 pixels, 1 pixel = 0.5112 µm × 0.5112 µm).

### HLA antibody testing and HLA typing

HLA antibody testing and HLA typing at our center have been recently described^27^. Antibody specificities were determined by LABScreen® Single Antigen Class I and Class II assay (One Lambda Inc., Canoga Park, CA). Single antigen beads were used to test for antibodies against HLA-A, -B, -C, -DRB1, -DRB3, -4 and -5, and -DQB1. Low-to-medium resolution HLA-A, -B, -C and DR typing was performed using BioSewoom™ PCR/SSP kit (BioSewoom Inc., Seoul, Korea) and high resolution HLA-DQB1 typing was done by AVITA™ plus HLA-DQB1 SBT kits (BioWithus Inc., Seoul, Korea).

### Clinical outcomes

The primary clinical outcome was death-censored graft failure (DCGF).

### Statistical analysis

Categorical variables are presented as absolute and relative frequencies. Quantitative variables are presented as mean and standard deviation (SD). Student’s t-test or Mann-Whitney U test was used to analyze differences between means, as appropriate. One-way analysis of variance was performed to compare the densities of intragraft NK cells according to histologic diagnosis. Categorical variables were compared using the chi-squared test. Area under the receiver operating characteristics curve (AUC) was measured to assess the ability of intragraft NK cells to differentiate histologic ABMR from the no rejection group and the TCMR group. Univariate and multivariate analyses were performed using the Cox proportional hazards model. Death-censored graft survival rate was calculated using the Kaplan-Meier method and compared using the log-rank test. A test for the proportionality property of hazards was performed for each factor considered within the multivariate model. P values < 0.05 were considered statistically significant. All statistical analyses were performed using SPSS version 21.0 for Windows (SPSS Inc., Chicago, IL, USA).

## Results

### Patient characteristics and histopathology

Table 1 shows the clinical and demographical characteristics of the 39 for-cause biopsies included in this study. The biopsies were divided into no rejection (NR; n = 8), T cell-mediated rejection (TCMR; n = 11), and antibody-mediated rejection (ABMR; n = 20) groups (Table 1). Nine patients with mixed rejection (TCMR + ABMR) were included in the ABMR group. As a total, the mean interval between kidney transplantation and for-cause biopsy was 23.1 ± 16.3 months. Compared with the NR group, the ABMR group had significantly longer mean interval between transplantation and biopsy (27.4 ± 16.7 vs. 10.5 ± 14.4 months, *P* = 0.03); there were no significant differences in terms of baseline characteristics between the two groups otherwise. In the TCMR group, borderline, type IA, and type IB rejections were diagnosed in 8 (72.7%), 2 (18.2%), and 1 (9.1%) specimens, respectively. Among the 9 patients with mixed rejections, type IA and type IB rejection were diagnosed in 4 (44.4%) and 5 (55.6%) specimens, respectively. Of the 20 patients with ABMR, acute and chronic active ABMR were confirmed in 14 (70%) and 6 (30%) specimens, respectively. IFTA was diagnosed in 32 specimens (82.0%), which were equally divided between mild and moderate-to-severe IFTA (Table 2). In terms of Banff score, the ABMR group had significantly higher mean i, ci, ct, and ti scores than did the NR group. The ABMR group had significantly higher mean g score compared with the TCMR group, and significantly higher mean ptc score compared with the other two groups. Steroid pulse therapy was administered in all patients with acute TCMR; one patient was steroid-resistant and was additionally administered with thymoglobulin. The primary treatment for ABMR was a combination of total plasma exchange, low-dose intravenous immunoglobulin, and rituximab.

**Table 1:** Clinical and demographic characteristics of patients with for-cause biopsy.

Variables	Total (n = 39)	NR (n = 8)	TCMR (n = 11)	ABMR (n = 20)	*P*-value
Mean age, y (SD)	43.7 (12.8)	45.5 (15.4)	46.0 (9.8)	42.6 (13.3)	0.735
Female sex, n (%)	8 (20.5)	2 (25.0)	1 (9.1)	5 (25.0)	0.789
Body mass index, kg/m^2^ (SD)	22.4 (2.7)	21.5 (3.7)	21.6 (2.7)	22.8 (2.4)	0.475
Deceased donor, n (%)	13 (33.3)	3 (37.5)	3 (27.3)	7 (35.0)	0.806
Previous transplant, n (%)	9 (23.1)	3 (37.5)	1 (9.1)	5 (25.0)	0.715
Time to biopsy, month (SD)	23.1 (16.3)	10.5 (14.4)	24.3 (12.9)	27.4 (16.7)	0.03
Cause of ESRD, n (%)					
Glomerular	5 (12.8)	2 (25.0)	0	3 (15.0)	
Diabetes	6 (15.4)	1 (12.5)	3 (27.3)	2 (10.0)	
Hypertension	4 (10.3)	2 (25.0)	0	2 (10.0)	
FSGS	1 (2.6)	1 (12.5)	0	0	
Other	17 (43.6)	2 (25.0)	6 (54.5)	9 (45.0)	
Unknown	6 (15.4)	0	2 (18.2)	4 (20.0)	
ABO-incompatible KT	10 (25.6)	3 (37.5)	3 (27.3)	4 (20.0)	0.625
Serum creatinine at the time of biopsy, mg/dL (SD)	2.7 (1.9)	2.7 (2.5)	2.3 (0.8)	2.9 (2.1)	0.746
PRA > 10%, n (%)	18 (46.2)	5 (62.5)	2 (18.2)	11 (55.0)	0.853
DSA at the time of biopsy, n (%)	22 (56.4)	4 (50.0)	3 (27.3)	15 (75.0)	0.088
CNI at the time of biopsy, n (%)					0.789
Cyclosporine	8 (20.5)	0	5 (45.5)	3 (15.0)	
Tacrolimus	31 (79.5)	8 (100)	6 (54.5)	17 (85.0)	
Follow-up after biopsy, month (SD)	46.7 (19.1)	38.1 (20.7)	49.1 (14.5)	48.8 (20.5)	0.317

NR, no rejection; TCMR, T-cell-mediated rejection; ABMR, antibody-mediated rejection; SD, standard deviation; ESRD, end-stage renal disease; FSGS, focal segmental glomerulosclerosis; KT, kidney transplantation; PRA, panel-reactive antibody; DSA, donor specific antibody; CNI, calcineurin inhibitor.

**Table 2:** Comparison of histopathologic characteristics according to histologic diagnosis.

Variables	Total (n = 39)	NR (n = 8)	TCMR (n = 11)	AmBR (n = 20)	*P* value
Histopathology, n (%)					0.009
TCMR only	11 (28.2)	0	11 (100)	0	
ABMR only	11 (28.2)	0	0	11 (55.0)	
ABMR + TCMR	9 (23.1)	0	0	9 (45.0)	
No rejection	8 (20.5)	8 (100)	0	0	
C4d staining, n (%)					0.747
<10%	34 (87.2)	7 (87.5)	9 (81.8)	18 (90.0)	
≥10%, <50%	5 (12.8)	1 (12.5)	2 (18.2)	2 (10.0)	
≥50%	0	0	0	0	
IFTA, n (%)					0.02
Minimal	7 (17.9)	4 (50.0)	1 (9.1)	2 (10.0)	
Mild	16 (41.0)	2 (25.0)	7 (63.6)	7 (35.0)	
Moderate-to-severe	16 (41.0)	2 (25.0)	3 (27.3)	11 (55.0)	
Mean Banff score, mean (SD)					
g	0.79 (0.83)	0.75 (1.04)	0.27 (0.47)	1.1 (0.79)	0.025
cg	0.31 (0.73)	0.13 (0.35)	0.09 (0.3)	0.5 (0.95)	0.246
mm	0.15 (0.37)	0.25 (0.46)	0.18 (0.41)	0.1 (0.31)	0.603
i	1.49 (1.07)	0.5 (0.76)	1.27 (0.91)	2.00 (0.97)	0.001
ci	1.26 (0.97)	0.5 (0.76)	1.18 (0.75)	1.6 (0.99)	0.019
t	1.46 (1.02)	0.75 (1.17)	1.64 (0.81)	1.65 (0.99)	0.084
ct	1.33 (0.90)	0.63 (0.74)	1.27 (0.65)	1.65 (0.93)	0.019
v	0.05 (0.22)	0.13 (0.35)	0	0.05 (0.22)	0.496
cv	1.05 (1.03)	0.63 (0.74)	1.27 (1.27)	1.1 (0.97)	0.389
ah	0.82 (1.02)	0.25 (0.46)	1.36 (1.21)	0.75 (0.97)	0.054
ptc	1.33 (1.18)	0.5 (1.07)	0.45 (1.04)	2.15 (0.59)	<0.001
ti	1.9 (0.97)	0.88 (0.64)	1.73 (0.65)	2.4 (0.88)	<0.001
i-IFTA	1.97 (0.99)	1.38 (1.30)	2.22 (0.97)	2.10 (0.79)	0.146

NR, no rejection; TCMR, T-cell-mediated rejection; ABMR, antibody-mediated rejection; IFTA, interstitial fibrosis and tubular atrophy; SD, standard deviation.

### Difference in the density of intragraft NK cells according to histologic diagnosis of kidney allograft

**Figure 1:**
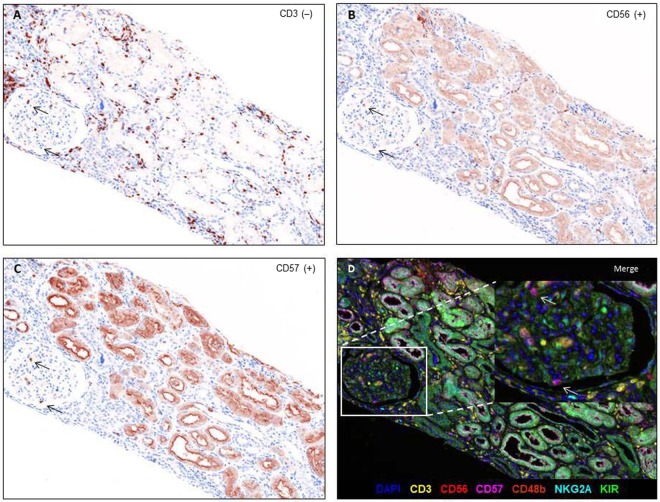
Multiplex immunohistochemistry of a kidney allograft with antibody-mediated rejection. Black arrows indicate CD3^−^ (**A**), CD56^+^ (**B**), CD57^+^ (**C**) lymphocytes in the glomerulus. Inset: higher-magnification view of a section demonstrating CD56^+^CD57^+^ NK cells in the glomerulus. Original magnification: ×200.

To characterize the subpopulations of intragraft NK cells, we performed multiplex immunohistochemistry on six markers (CD3, CD56, CD57, CD49b, NKG2A, and KIR). Intragraft NK cells were observed in peritubular capillaries as well as in interstitial spaces (Fig. 1), and the density of intragraft NK cells was relatively low compared with that of infiltrated CD3^+^ mononuclear cells.

**Figure 2:**
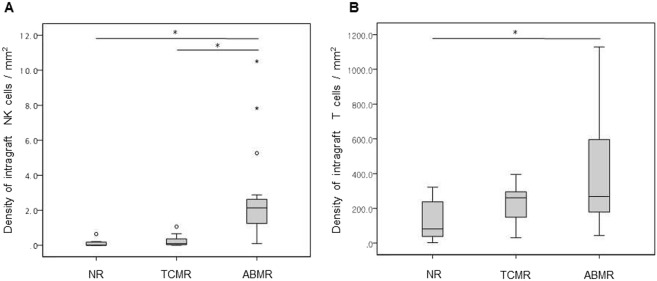
Density of intragraft NK (**A**) and T (**B**) cells according to histologic diagnosis. NR, no rejection; TCMR, T cell-mediated rejection; ABMR, antibody-mediated rejection. *P < 0.01.

**Figure 3:**
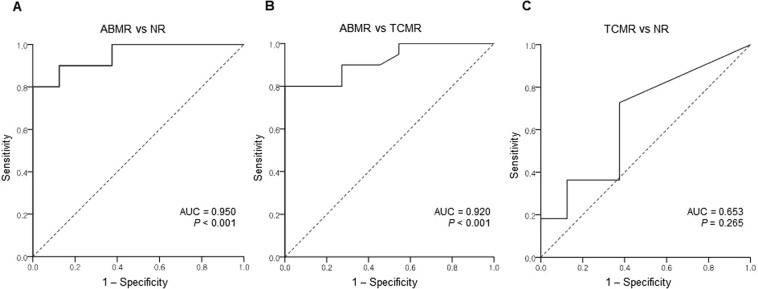
Logistic regression analysis on the association between ABMR and the density of NK cell infiltration. AUC, area under the curve; NR, no rejection; TCMR, T cell-mediated rejection; ABMR, antibody-mediated rejection.

**Figure 4:**
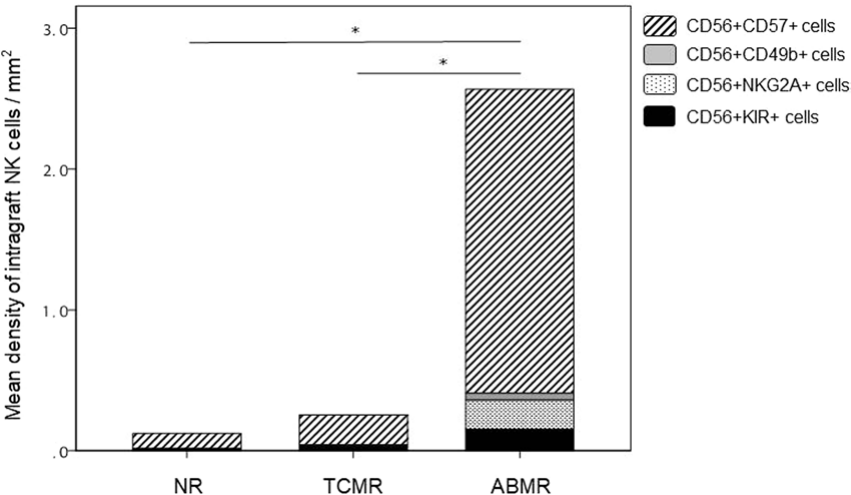
Mean density of intragraft NK cells according to histologic diagnosis. NR, no rejection; TCMR, T cell-mediated rejection; ABMR, antibody-mediated rejection. *P < 0.05, a statistical significance in comparing the density of CD56^+^ CD57^+^ cells between three groups.

Densities of intragraft NK and T cells were compared according to histologic diagnosis using one-way analysis of variance. The density of intragraft NK cells was significantly higher in the ABMR group (2.57 ± 2.58/mm^2^) compared with the NR (0.12 ± 0.22/mm^2^) and the TCMR (0.25 ± 0.34/mm^2^) groups (*P* = 0.002) (Fig. 2A). Furthermore, the density of intragraft T cells was significantly higher in the ABMR group (393.40 ± 300.93/mm^2^) compared with the NR (129.83 ± 119.09/mm^2^) (Fig. 2B). It is likely that the density of NK infiltrates is correlated with that of T cells only in the ABMR group with marginal significance (r = 0.4000, *P* = 0.081). When we correlated the density of NK cells with the phenotypes of for-cause biopsy, the density of intragraft NK cells effectively differentiated ABMR from the TCMR (AUC = 0.95, *P* < 0.001) and the NR (AUC = 0.92, *P* < 0.001) groups with good discriminative performance (Fig. 3). We also evaluated the subpopulations of intragraft NK cells according to histologic diagnosis, and found that CD56^+^CD57^+^ infiltrates were the most predominant subpopulation of intragraft NK cells in the ABMR group (Fig. 4). When one-way analysis of variance was performed to compare the density of CD56^+^CD57^+^ cells between three histologic groups, the density was significantly higher in the ABMR group compared with the NR (p = 0.003) and the TCMR (p = 0.002). In the ABMR group, furthermore, CD56^+^CD57^+^ infiltrates (2.16 ± 1.89/mm^2^) were the most frequently observed NK cells compared with CD56^+^CD49b^+^ (0.05 ± 0.13/mm^2^), CD56^+^NKG2A^+^ (0.21 ± 0.69/mm^2^), and CD56^+^KIR^+^ (0.15 ± 0.42/mm^2^) cells in the ABMR group (*P* < 0.001) when paired t-test was performed. Interestingly, the infiltration of CD56^+^NKG2A^+^ cells was observed only in the ABMR group although the density (0.21 ± 0.69/mm^2^) is low. Meanwhile, CD56^+^CD49b^+^ and CD56^+^KIR^+^ cells were infiltrated predominantly in the ABMR group although there was no statistical significance.

### NK cell infiltration and histological lesions and DSA

**Figure 5:**
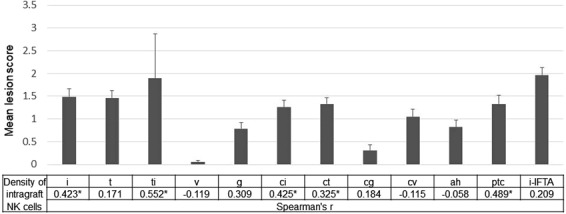
Association between the density of intragraft NK cells and histologic lesions. Mean lesion scores were calculated for each listed histologic lesion (i, interstitial inflammation; t, tubulitis; v, intimal arteritis; ptc, peritubular capillaritis; g, glomerulitis; cg, transplant glomerulopathy; ci, interstitial fibrosis; ct, tubular atrophy). Corresponding Spearman’s correlation coefficients between the density of intragraft NK cells and the listed histologic lesion scores are shown. *P < 0.05.

We investigated the correlation between the density of intragraft NK cells and histological lesions scores. Pearson correlation calculation showed that the density of intragraft NK cells was significantly correlated with i (r = 0.423, *P* = 0.007), ti (r = 0.552, *P* < 0.001), ci (r = 0.425, *P* = 0.007), ct (r = 0.325, *P* = 0.044), and ptc (r = 0.489, *P* = 0.002) scores whereas g score (r = 0.309, *P* = 0.056) was associated with the density of intragraft NK cells with marginal significance (Fig. 5). It is likely that the mean fluororescence intensity (MFI) of DSA was correlated with the frequency of intragraft NK cells (r = 0.317, *P* = 0.050) as well as intragraft CD56^+^CD57^+^ cells (r = 0.294, *P* = 0.07), however, without a statistical significance.

### Impact of NK cell infiltration on kidney allograft failure

**Figure 6:**
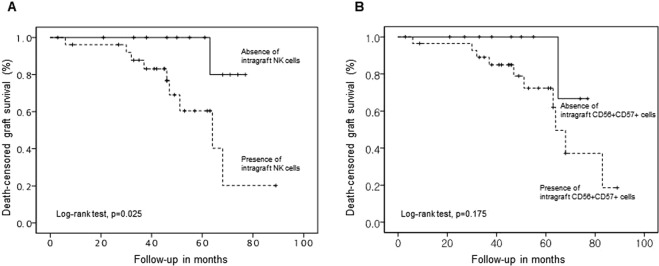
Kaplan-Meier curve for 8-year death-censored kidney allograft survival according to the presence of intragraft NK cells (**A**) and the presence of CD56^+^ CD57^+^ cells (**B**).

The impact of baseline characteristics and histological factors on death-censored graft failure after for-cause biopsy was assessed by univariable Cox regression analysis, and the resultant significant factors were entered into a multivariable model (Table 3). Multivariable analyses revealed that presence of intragraft NK cells (adjusted hazard ratio (AHR), 10.6; 95% confidence interval (CI), 1.01–110.82; *P* = 0.049), serum creatinine at the time of biopsy (AHR, 1.66; 95% CI, 1.12–2.45; *P* = 0.012), and diabetes (AHR, 11.68; 95% CI, 1.85–73.85; *P* = 0.009) were independently associated with death-censored graft failure after for-cause biopsy. Importantly, recipients with intragraft NK cells had a significantly higher rate of death-censored graft failure compared with those without intragraft NK cells (*P* = 0.025, Fig. 6A). Although there was no significant difference in death-censored graft survival between the two groups, the death-censored graft survival in those with intragraft CD56^+^CD57^+^ NK cells tends to be lower compared with those without those cells (Fig. 6B).

**Table 3:** Death-censored graft failure and adjusted HR from multivariate Cox regression.

Variables	HR_unadj_ (95% CI)	HR_adj_ (95% CI)	*P*-value
Presence of intragraft NK cells	7.81 (0.97–63.18)	10.60 (1.01–110.82)	0.049
Serum creatinine at the time of biopsy, mg/dL	1.38 (1.03–1.86)	1.66 (1.12–2.45)	0.012
Chronic active ABMR	4.03 (1.13–14.32)	1.01 (0.21–4.83)	0.986
Diabetes	3.45 (0.97–12.3)	11.68 (1.85–73.85)	0.009

HR_unadj_, unadjusted hazard ratio; CI, confidence interval; HR_adj,_ adjusted hazard ratio; NK, natural killer; ABMR, antibody-mediated rejection; HPF, high-power field.

## Discussion

In this study, we found that patients diagnosed as ABMR had higher densities of NK cells in for-cause biopsies. Notably, CD56^+^CD57^+^ infiltrates were the most predominant subset among the intragraft NK cells. Additionally, we demonstrated that NK cell infiltration in kidney allografts was associated with poor death-censored graft survival. To our knowledge, this study is one of the few that used the multiplex immunohistochemistry technique to demonstrate an association between high density of overall NK cell infiltration and ABMR. Notably, we further identified the specific subset of intragraft NK cells (CD3^−^CD56^+^CD57^+^) that were related to ABMR, which was confirmed in the multiplex immunohistochemistry.

Previous studies have reported the association between intrarenal NK cell transcripts with ABMR. Hidalgo *et al*. verified a distinct role of NK cells in late ABMR as well as a role for NK-transcript expressing cells in both TCMR and inflammation-associated injury and atrophy scarring^13,28^. Venner *et al*. used microarray analysis of kidney allograft with ABMR to show that NK cells are involved in ABMR through CD16a Fc receptors^12^. Similarly, Yazdani *et al*. demonstrated that ABMR is associated with an intrarenal expression signature enriched with NK cell pathways^11^; however, in contrast to other studies, the authors reported that activated the NK cells are the only cell types that discriminate ABMR from TCMR and have predictive value for transplant outcomes. In their study, Yazdani *et al*. only used a single phenotypic marker (NKp46) to identify intragraft NK cells and analyzed only nine cases of ABMR^11^. On the other hand, to better identify the specific subpopulations of intragraft NK cells, we used Opal™ multiplex kit to simultaneously analyze six markers (CD3, CD56, CD57, CD49b, NKG2A, and KIR) in each FFPE tissue section from 20 cases of ABMR. As a result, we clearly observed that CD3^−^CD56^+^CD57^+^ NK cells in the for-cause biopsy specimens were significantly associated with ABMR.

In our study, CD56^+^CD57^+^ NK cells were the predominant subpopulation in kidney allografts of ABMR cases. The CD57 antigen is a 100–115 kD terminally sulfated carbohydrate epitope that was originally reported as a marker of human natural killer cells. While CD57 expression on human lymphocytes indicates an inability to proliferate, these cells also display high cytotoxic potential while exhibiting both memory-like features and potent effector functions^29^. Interestingly, it has been reported that infection with viruses including human immunodeficiency virus and cytomegalovirus could drive the expansion of CD57^+^ NK cells^30,31^. Additionally, increased expression of CD16 by CD57^+^CD56^dim^CD16^+^ NK cells may render them hyper-responsive to CD16 ligation, which mimics antibody-dependent cell-mediated cytotoxicity (ADCC). Mature NK cells exhibit considerable cytotoxic potential as indicated by elevated expression of the degranulation marker CD107a, granzyme B, and perforin^29^. It has been reported that NK cells are involved in ABMR through CD16a Fc receptors triggering ADCC^12^, but it is yet unclear how CD57^+^ NK cells interact with CD16a for inducing ADCC. As well as CD56^+^CD57^+^ NK cells, other subsets of intragraft NK cells were predominant in the ABMR group although the density of each subset was relatively lower compared with CD56^+^CD57^+^ NK cells. According to previous reports, it is known that CD56^+^NKG2A^+^ and CD56^+^KIR^+^ cells are supposed to inactivate NK cells interacting with HLA-E and distinct sets of classical HLA class I molecules^19–21^. It is necessary to determine whether CD56^+^NKG2A^+^ and CD56^+^KIR^+^ counteract the effects of CD56^+^CD57^+^ NK cells by functional studies in the near future. On the other hand, it has been known that CD49b is one of maturation-associated molecules of conventional NK cell^32–35^. In addition, recent studies reported that CD49a^+^CD49b^−^ NK cells are tissue-resident in liver, uterus, and skin^36,37^. It has not been elucidated yet whether CD56^+^CD49b^+^ NK cells have differential characteristics compared to CD56^+^CD49b^−^ NK cells in human kidney allograft.

We observed that the TCMR group also had CD56^+^CD57^+^ NK cells in the kidney allografts, although the proportion was significantly lower compared with those in the ABMR group. Considering that borderline rejection was predominant in the TCMR group and that about 27 percent recipients in the TCMR group had DSA at the time of biopsy, it is possible that there was antibody-mediated injury in the TCMR group without a pathologic evidence. On the other hand, this is in line with the results of a previous study, which demonstrated that CD16a-activated NK cells in ABMR and CD3/TCR-activated T cells in TCMR are highly similar in terms of rejection pathogenesis^38^.

This study is limited in that because there were relatively few cases of ABMR available for analysis, and that a portion of the ABMR group had mixed phenotype of TCMR and ABMR. Nevertheless, the mixed TCMR + ABMR cases had a significantly higher density of infiltrating NK cells compared with the TCMR group, thereby justifying their inclusion in the ABMR group. Also, due to the small sample size and the single-center design of the study, we could not designate a definite cut-off value for the density of NK cell infiltration for discriminating among different clinical outcomes. In addition, infiltrates in five samples were positive for more than three out of four NK markers (CD57, CD49b, NKG2A and KIR). For those samples, therefore, infiltrates were counted repeatedly and divided by the number of positive markers to belong to each group in Fig. 4. Nevertheless, densities of CD56^+^CD49b^+^, CD56^+^NKG2A^+^, and CD56^+^KIR^+^ were much lower compared with the CD56^+^CD57^+^ group. Initially, we intended to investigate clinical implications of each NK cell subset with respect to graft survival. However, relevant analysis was insufficient due to the low density of some NK subsets as well as the small sample size of this study. We think that it is important to understand mechanistic and functional significances of co-expression of NK markers. For those purposes, however, it is necessary to perform *in vitro* study with cell lines in the near future. In spite of several limitations, this study is worthy in that it revealed the association between NK cell infiltration and poor clinical outcomes. We already reported that higher numbers of CD56^+^ cell infiltration in for-cause biopsy was associated with poor clinical outcomes^27^. A prospective multi-center study should be carried out in order to evaluate the long-term clinical implications of NK cell infiltration observed in protocol biopsy as well as in for-cause biopsy. Finally, the exact mechanisms by which NK cells influence ABMR or ADCC was not investigated. This study suggests that CD57 is an important mediator between NK cells and ABMR. Therefore, comprehensive *in vitro* and *in vivo* studies are necessary in order to delineate the role of CD57 in NK cell-mediated graft injury. In this regard, a combination of multiplex immunohistochemistry and microarray transcription analysis would be useful for more detailed characterization and analysis of intragraft NK cells.

In conclusion, we demonstrated that the presence of NK cells in for-cause biopsy of kidney allografts was significantly associated with ABMR and poor graft survival. It is noteworthy that CD56^+^CD57^+^ infiltrates were the most predominant subset of intragraft NK cells in renal transplant biopsies with ABMR. Further studies are needed to determine the mechanism of NK cell infiltration in kidney allografts of patients with ABMR.

## Data Availability

Raw data is securely stored in the Data Base of Asan Institute for Life Sciences and available following an official permission from the corresponding author.
